# Prognostic value of pre-irradiation FET PET in patients with not completely resectable IDH-wildtype glioma and minimal or absent contrast enhancement

**DOI:** 10.1038/s41598-021-00193-x

**Published:** 2021-10-21

**Authors:** Jurij Rosen, Gabriele Stoffels, Philipp Lohmann, Elena K. Bauer, Jan-Michael Werner, Michael Wollring, Marion Rapp, Jörg Felsberg, Martin Kocher, Gereon R. Fink, Karl-Josef Langen, Norbert Galldiks

**Affiliations:** 1 Department of Neurology, Faculty of Medicine, University Hospital Cologne, University of Cologne, Kerpener Str. 62, 50937 Cologne, Germany; 2 Institute of Neuroscience and Medicine (INM-3, -4), Research Center Juelich, Juelich, Germany; 3 Department of Stereotaxy and Functional Neurosurgery, Faculty of Medicine and University Hospital Cologne, University of Cologne, Cologne, Germany; 4 Department of Neurosurgery, University Hospital Duesseldorf, Duesseldorf, Germany; 5 Institute of Neuropathology, University Hospital Duesseldorf, Duesseldorf, Germany; 6 Department of Nuclear Medicine, University Hospital Aachen, Aachen, Germany; 7 Center for Integrated Oncology (CIO), Universities of Aachen, Bonn, Cologne, and Duesseldorf, Germany

**Keywords:** CNS cancer, Cancer in the nervous system

## Abstract

In glioma patients, complete resection of the contrast-enhancing portion is associated with improved survival, which, however, cannot be achieved in a considerable number of patients. Here, we evaluated the prognostic value of *O*-(2-[^18^F]-fluoroethyl)-L-tyrosine (FET) PET in not completely resectable glioma patients with minimal or absent contrast enhancement before temozolomide chemoradiation. Dynamic FET PET scans were performed in 18 newly diagnosed patients with partially resected (n = 8) or biopsied (n = 10) IDH-wildtype astrocytic glioma before initiation of temozolomide chemoradiation. Static and dynamic FET PET parameters, as well as contrast-enhancing volumes on MRI, were calculated. Using receiver operating characteristic analyses, threshold values for which the product of paired values for sensitivity and specificity reached a maximum were obtained. Subsequently, the prognostic values of FET PET parameters and contrast-enhancing volumes on MRI were evaluated using univariate Kaplan–Meier and multivariate Cox regression (including the MTV, age, MGMT promoter methylation, and contrast-enhancing volume) survival analyses for progression-free and overall survival (PFS, OS). On MRI, eight patients had no contrast enhancement; the remaining patients had minimal contrast-enhancing volumes (range, 0.2–5.3 mL). Univariate analyses revealed that smaller pre-irradiation FET PET tumor volumes were significantly correlated with a more favorable PFS (7.9 vs. 4.2 months; threshold, 14.8 mL; *P* = 0.012) and OS (16.6 vs. 9.0 months; threshold, 23.8 mL; *P* = 0.002). In contrast, mean tumor-to-brain ratios and time-to-peak values were only associated with a longer PFS (*P* = 0.048 and *P* = 0.045, respectively). Furthermore, the pre-irradiation FET PET tumor volume remained significant in multivariate analyses (*P* = 0.043), indicating an independent predictor for OS. Our results suggest that pre-irradiation FET PET parameters have a prognostic impact in this subgroup of patients.

## Introduction

Astrocytic gliomas represent a pheno- and genotypically defined group of central nervous system neoplasms characterized by a rapid and infiltrative growth^1^. Despite the availability of a standardized treatment comprising surgery followed by chemoradiation with alkylating agents, the patients’ prognosis remains poor. This poor prognosis particularly applies to astrocytic glioma patients without an isocitrate dehydrogenase (IDH) mutation and an only incompletely resectable tumor due to its localization in deep or eloquent brain areas. Furthermore, the recent interim analysis of the CATNON trial suggests that in patients with IDH-wildtype astrocytic gliomas, radiotherapy combined with maintenance temozolomide chemotherapy is of limited efficacy^2^.

In the diagnostic workup of patients with glioma, contrast-enhanced MRI has a pivotal role in detecting, characterizing, and planning surgical tumor resection. After resection, presence of contrast enhancement on the early postoperative MRI within 24–72 h is assumed to indicate residual tumor, i.e., an incomplete resection^3^. Notably, a complete versus only partial resection according to these criteria has a relevant impact on the patient’s prognosis^4–7^. However, a considerable number of patients especially with IDH-wildtype anaplastic glioma lack contrast enhancement on MRI^8,9^, so this parameter cannot be used for resection guidance and assessment. Thus, in this patient group, the limited information about the extent of the tumor tissue to be resected may contribute to the poor survival prognosis. Hence, additional neuroimaging techniques are warranted.

In this context, PET using radiolabeled amino acids is an alternative that allows delineating the tumor extent more precisely^10,11^. Especially in Europe, the radiolabeled amino acid *O*-(2-[^18^F]fluoroethyl)-L-tyrosine (FET) is currently the most frequently used tracer^12^. The main advantage of PET using radiolabeled amino acids is that the uptake of these tracers is independent of blood–brain barrier disruption and therefore detects tumor parts not showing contrast enhancement on MRI^13,14^.

Moreover, FET PET has been shown to harbor prognostic value already at an early disease stage. For instance, static and dynamic FET PET parameters identified subgroups with a more favorable prognosis in patients with newly diagnosed IDH-wildtype glioma^15^, or postoperatively, i.e., before initiation of temozolomide chemoradiation^16,17^. In contrast to the present work, the patients evaluated in these studies had predominantly contrast-enhancing gliomas and clearly higher rates of complete resections. Here, we retrospectively identified prognostically unfavorable patients with non-completely resectable, IDH-wildtype astrocytic glioma with minimal or absent contrast enhancement on MRI. To identify a subgroup with improved progression-free and overall survival (PFS, OS), we evaluated the prognostic value of static and dynamic FET PET parameters before initiation of chemoradiation with temozolomide.

## Patients and methods

### Patients

From 2013–2019, we retrospectively identified patients who (i) were diagnosed with newly diagnosed and histomolecularly characterized IDH-wildtype astrocytic glioma not eligible for complete resection, showed (ii) minimal (i.e., ≤ 5 mL) or absent MRI contrast enhancement, and (iii) had undergone MR and FET PET imaging before initiation of radiotherapy.

According to these search criteria, we identified 18 adult patients (mean age, 51 ± 14 years; age range, 24–66 years; 6 females). Due to tumor localization in deep or eloquent brain areas, ten patients underwent stereotactic biopsy. In the remaining eight patients, only partial resection could be achieved. The patients either had no contrast enhancement (n = 8) or minimal contrast enhancement on MRI (n = 10). FET PET imaging was performed 17 ± 16 days prior to biopsy or partial resection.

Seventeen of 18 patients were treated according to the EORTC/NCIC 22,981/26,981 trial with radiotherapy and concomitant temozolomide chemotherapy followed by maintenance temozolomide chemotherapy over six cycles^18^. Fourteen patients completed radiotherapy with concomitant and maintenance temozolomide chemotherapy over six cycles. One patient refused chemotherapy and was treated with radiotherapy only.

During follow-up, contrast-enhanced conventional MRI was performed every 8–12 weeks. Furthermore, patients were assessed by neurological examination, and the Karnofsky Performance Score was determined every 8–12 weeks during the treatment and after treatment completion. The patients’ outcome was evaluated by calculating the PFS and OS. The PFS was defined as the time interval between histomolecularly confirmed glioma diagnosis and tumor progression according to the RANO criteria^19^. The OS was defined as the time interval between histomolecularly confirmed glioma diagnosis and death. The median follow-up time was 13.7 months (range 6.5–31.4 months). Table 1 provides a summary of the patients’ characteristics.

**Table 1:** Patient characteristics.

#	Gender	Age at diagnosis	MGMT promoter methylation	IDH mutation	Diagnosis	WHO grade	Tumor localization	Extent of resection	RT	PFS (months)	OS (months)
1	F	50	Meth	wt	glioblastoma	IV	temporal right	B	RT + TMZ	6.6	8.0
2	M	29	n.d	wt	H3K27M	IV	mesencephalon left	B	RT + TMZ	7.8	13.3
3	M	65	Meth	wt	astrocytoma	III	thalamus left	PR	RT + TMZ	7.3	26.5
4	M	66	Meth	wt	glioblastoma	IV	insula right	B	RT + TMZ	15.7	28.9
5	F	48	Not meth	wt	glioblastoma	IV	parietal left	B	RT + TMZ	2.1	31.4
6	F	24	Not meth	wt	astrocytoma	III	thalamus left	B	RT + TMZ	6.7	14.0
7	M	51	Not meth	wt	glioblastoma	IV	parietal left	PR	RT + TMZ	4.1	7.0
8	M	30	Meth	wt	astrocytoma	III	frontal left	PR	RT + TMZ	6.0	15.9
9	M	42	Meth	wt	astrocytoma	II	temporal left	B	RT + TMZ	6.0	11.6*
10	M	56	Not meth	wt	glioblastoma	IV	temporal right	PR	RT + TMZ	10.5	14.0
11	F	34	Not meth	wt	glioblastoma	IV	parietal left	PR	RT + TMZ	5.7	6.5*
12	M	62	Meth	wt	glioblastoma	IV	parietal left	PR	RT + TMZ	4.2	10.0
13	M	54	Not meth	wt	glioblastoma	IV	parietal right	PR	RT + TMZ	3.3	7.7
14	F	66	n.d	wt	astrocytoma	II	temporal left	B	RT alone	7.9	11.4
15	M	58	Not meth	wt	glioblastoma	IV	temporal left	B	RT + TMZ	9.3	12.0
16	M	50	Not meth	wt	astrocytoma	n.d	insula left	B	RT + TMZ	9.3	21.8
17	F	41	Not meth	wt	glioblastoma	IV	parietal left	PR	RT + TMZ	3.7	16.9
18	M	66	Meth	wt	astrocytoma	III	bithalamic	B	RT + TMZ	11.3	16.2

*B* biopsy, *F* female, *H3K27* H3K27-mutant diffuse midline glioma, *IDH* isocitrate dehydrogenase, *M* male, *meth/not* *meth* MGMT promoter methylated / not methylated, *MGMT* O^6^-methylguanine DNA methyltransferase, *n.d.* not determined, *OS* overall survival, *PFS* progression-free survival, *PR* partial resection, *RT* radiotherapy, *TMZ* temozolomide, *wt* wildtype, * censored.

### MR imaging

Following the International Standardized Brain Tumor Imaging Protocol (BTIP)^20^, MR imaging was performed using a 1.5 T or 3.0 T MRI scanner with a standard head coil before and after administration of a gadolinium-based contrast agent (0.1 mmol/kg body weight). The sequence protocol comprised 3D isovoxel T1-weighted, 2D T2-weighted, and 2D fluid-attenuated inversion recovery-weighted (FLAIR) sequences. Volumes of contrast enhancement and non-enhancing FLAIR-signal abnormality were automatically segmented using the HD-GLIO brain tumor segmentation tool^21,22^. The automatic segmentation results were visually validated and manually revised, if necessary, using the software PMOD (Version 3.9, PMOD Technologies Ltd., Zurich, Switzerland).

### FET PET imaging

As described previously, the amino acid FET was produced via nucleophilic ^18^F-fluorination with a radiochemical purity of greater than 98%, specific radioactivity greater than 200 GBq/µmol, and a radiochemical yield of about 60%^23^. According to national and international guidelines for brain tumor imaging using labeled amino acid analogs^24^, all patients fasted for at least four hours before the PET measurements. All patients underwent a dynamic PET scan from 0 to 50 min post-injection of 3 MBq of FET per kg of body weight. PET imaging was performed either on an ECAT Exact HR + PET scanner (n = 7 patients) in 3-dimensional mode (Siemens, Erlangen, Germany) (axial field-of-view, 15.5 cm) or simultaneously with 3 T MR imaging using a BrainPET insert (n = 11 patients) (Siemens, Erlangen, Germany). The BrainPET is a compact cylinder that fits into the bore of the Magnetom Trio MR scanner (axial field of view, 19.2 cm)^25^.

Iterative reconstruction parameters were 16 subsets, six iterations using the OSEM algorithm for ECAT HR + PET scanner and two subsets, 32 iterations using the OP-OSEM algorithm for the BrainPET. Data were corrected for random, scattered coincidences, dead time, and motion for both systems. Attenuation correction for the ECAT HR + PET scan was based on a transmission scan, and for the BrainPET scan on a template-based approach^25^. The reconstructed dynamic data set consisted of 16 time frames (5 × 1 min; 5 × 3 min; 6 × 5 min) for both scanners.

To optimize comparability of the results related to the influence of the two different PET scanners, reconstruction parameters, and post-processing steps, a 2.5 mm 3D Gaussian filter was applied to the BrainPET data before further processing, resulting in an image resolution of approximately 4 mm (image resolution of the ECAT HR + PET scanner, approximately 6 mm). In phantom experiments using spheres of different sizes to simulate lesions, this filter kernel demonstrated the best comparability between PET data obtained from the ECAT HR + PET and the BrainPET scanner^26^.

### FET PET data analysis

FET PET data analysis was performed as described previously^27^. In brief, for the evaluation of FET data, summed PET images over 20–40 min post-injection were used. Mean amino acid uptake in the tumor was determined by a 2-dimensional auto-contouring process using a tumor-to-brain ratio (TBR) of 1.6 as described previously^9,28^. For calculating the maximal amino acid uptake, a circular ROI with a diameter of 1.6 cm was centered on the maximal tumor uptake^27^. Maximum and mean TBRs (TBR_max_, TBR_mean_) were calculated by dividing the maximum and mean standardized uptake value (SUV) of the tumor ROIs by the mean SUV of a larger ROI placed in the contralateral unaffected hemisphere including both gray and white matter as recommended by international guidelines^24^. The FET metabolic tumor volume (MTV) was determined by a 3-dimensional auto-contouring process using a TBR of 1.6 or more using the software PMOD (Version 3.9, PMOD Technologies Ltd., Zurich, Switzerland).

As described previously^27^, time-activity curves (TAC) of FET uptake in the tumor were generated by applying a spherical volume-of-interest (VOI) with a volume of 2 mL centered on the maximal tumor uptake to the entire dynamic dataset. A reference TAC was generated by placing a reference ROI in the unaffected brain tissue (as described above). For TAC evaluation, the time-to-peak (TTP; defined as the time in minutes from the beginning of the dynamic acquisition up to the lesion’s maximum SUV) was calculated. In cases with constantly increasing FET uptake without identifiable peak uptake, we defined the end of the dynamic PET acquisition as TTP. Furthermore, the TAC slope in the late phase of FET uptake was assessed by fitting a linear regression line to the late phase of the curve (20–50 min post-injection). The slope was expressed as the change of the SUV per hour. This procedure enables a more objective evaluation of kinetic data than a TAC assignment to FET uptake patterns^27^.

### Neuropathological tumor classification and analysis of molecular markers

All tumors were histomolecularly classified according to the World Health Organization (WHO) Classification of Tumors of the Central Nervous System of 2016^1^. For molecular biomarker analysis, tumor DNA was extracted from formalin-fixed and paraffin-embedded tissue samples with a histologically estimated tumor cell content of 80% or more. For assessing the isocitrate dehydrogenase (IDH) mutation status, the presence of an IDH1-R132H mutation was evaluated by immunohistochemistry using a mutation-specific antibody in a standard immunohistochemical staining procedure as reported^29,30^. If immunostaining for IDH1-R132H remained negative, the mutational hot-spots at codon 132 of IDH1 and codon 172 of IDH2 were directly sequenced as reported^31,32^. The MGMT promoter methylation status was assessed by methylation-specific PCR, as described elsewhere^32^.

### Statistical analysis

Descriptive statistics are provided as mean and standard deviation or median and range. The prognostic value of the FET PET parameters (TBR_max_, TBR_mean_, and MTV), as well as dynamic FET PET parameters (TTP, slope), was assessed by receiver operating characteristic (ROC) curve analyses using a favorable PFS and OS as reference. A favorable outcome was defined as a PFS ≥ 7.0 months and an OS ≥ 15.0 months, similar to the survival reported in the EORTC-NCIC 22,981/26,981 trial (PFS, 6.9 months; OS, 14.6 months)^18^. Thus, slightly higher values for PFS and OS were considered as favorable outcome thresholds. Decision cut-off was considered optimal when the product of paired values for sensitivity and specificity reached its maximum. When this product was identical for different thresholds, the threshold resulting in the best survival estimate was selected. As a measure of the test’s diagnostic quality, the area under the ROC curve (AUC), its standard error, and significance level were determined. Only patients with uncensored survival data were included in ROC analyses for the evaluation of the diagnostic performance, i.e., all patients (n = 18) for PFS, and 16 patients for OS. Univariate survival analyses were performed using Kaplan–Meier estimates. The log-rank test was used for comparison of the median PFS and OS between the subgroups. Multivariate Cox proportional hazards models were constructed to test the relationship between MTV and other clinical parameters (i.e., age, contrast-enhancing volume on MRI, and MGMT promoter methylation) for survival prediction. Hazard ratios (HR) and their 95%-confidence intervals (CI) were calculated. *P*-values of 0.05 or less were considered statistically significant. For statistical analyses and creation of figures R software was used^33^.

### Ethics approval

The local ethics committee of the RWTH University Aachen approved the retrospective analysis of the neuroimaging data. The study is in accordance with the declaration of Helsinki.

### Consent to participate

Before PET imaging, all subjects had given written informed consent for the PET and MRI investigation.

### Consent for publication

All subjects gave written informed consent for the use of the clinical data for scientific purposes.

## Results

### Patients

The histomolecularly confirmed initial diagnoses were distributed as follows: WHO grade II diffuse astrocytoma (n = 2), WHO grade III anaplastic astrocytoma (n = 4), WHO grade IV glioblastoma (n = 10), WHO grade IV H3K27M-mutated midline glioma (n = 1), and a WHO grade not specified pleomorphic astrocytoma (n = 1). All patients had an IDH wildtype, and seven patients had a methylated MGMT promotor (39%). In two patients, the MGMT promoter status could not be determined. In the whole cohort, the median PFS was 6.7 months (range 2.1–15.7 months), and the median OS was 13.7 months (range 6.5–31.4 months). Patient characteristics and neuroimaging findings are listed in Tables 1 and 2.

**Table 2:** Imaging findings.

#	TBR_max_	TBR_mean_	MTV (mL)	TTP (minutes)	Slope (SUV/h)	Contrast-enhancing volume on MRI (mL)	FLAIR volume on MRI (mL)
1	2.0	1.8	27.4	28	− 1.0	1.8	85.9
2	0.9	0.9	0.0	n.a	n.a	0.0	22.7
3	1.9	1.9	1.2	33	− 0.4	3.7	34.6
4	2.7	2.0	13.8	28	− 0.1	0.0	48.7
5	3.6	2.4	22.4	16	− 1.0	5.3	13.7
6	2.9	2.2	41.0	13	− 1.2	1.4	73.5
7	2.0	1.6	39.0	10	− 1.0	0.2	41.9
8	2.7	2.0	10.3	43	2.0	0.0	101.4
9	1.7	1.7	1.9	19	− 0.2	0.2	40.2
10	3.1	2.1	30.6	38	− 0.1	2.6	62.2
11	2.9	2.1	27.9	10	− 2.3	0.2	21.5
12	2.6	2.2	25.2	33	0.3	1.1	8.4
13	3.2	2.1	50.3	33	0.1	0.0	27.9
14	1.8	1.8	0.9	33	− 0.6	0.0	37.7
15	1.9	1.9	1.1	43	0.4	1.1	77.9
16	1.0	1.0	0.0	n.a	n.a	0.0	33.7
17	2.4	2.1	15.7	38	0.4	0.0	11.7
18	1.8	1.8	2.8	38	0.6	0.0	25.8

*FLAIR* fluid attenuated inversion recovery, *MTV* metabolic tumor volume, *n.a.* not available, *slope* slope of tracer uptake 20–50 min post-injection*,* *SUV* standardized uptake value, *TBR*_*max*_ maximum tumor-to-brain ratio, *TBR*_*mean*_ mean tumor-to-brain ratio, *TTP* time-to-peak.

### Optimal thresholds derived from FET PET and MRI parameters

**Figure 1:**
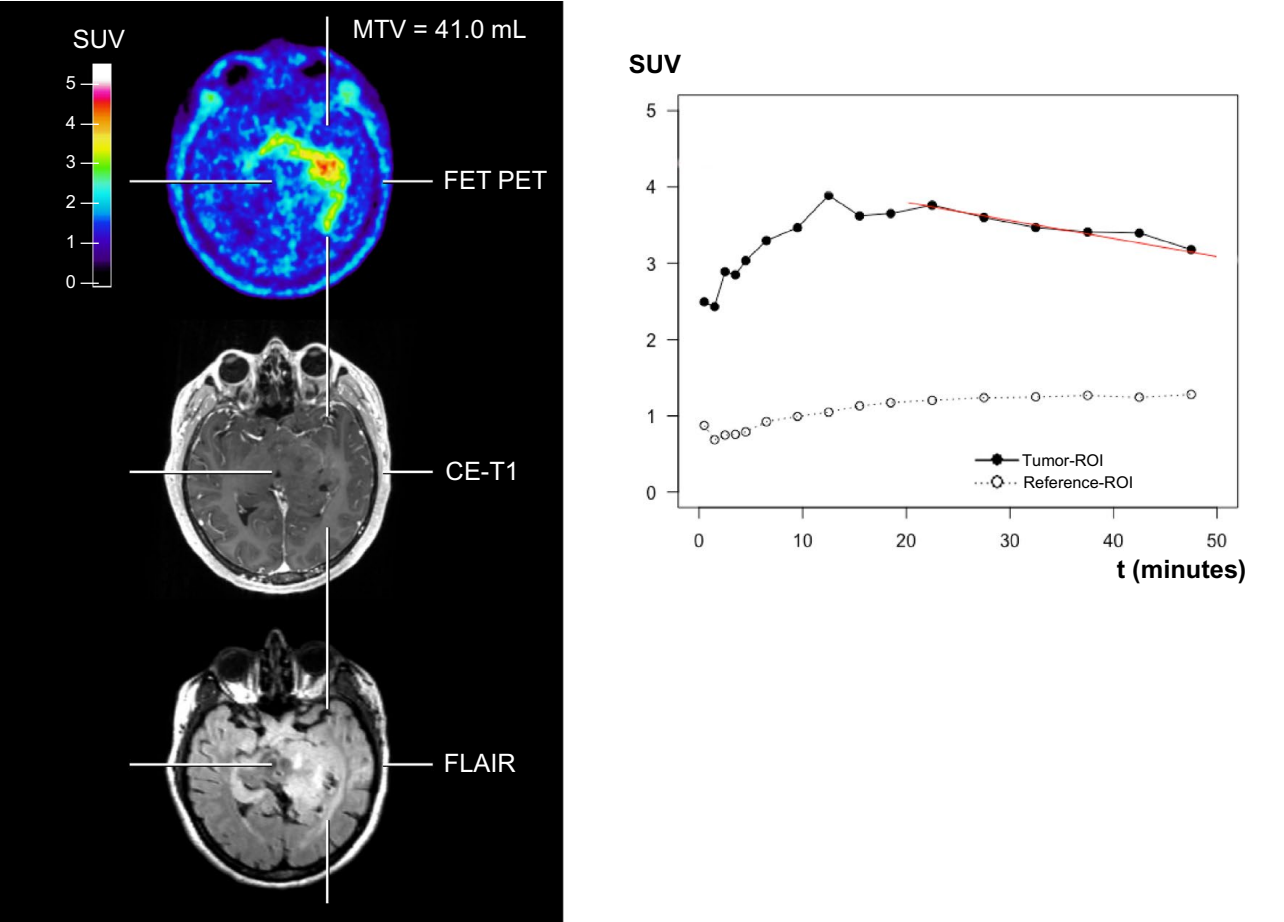
Representative neuroimages including FET PET, contrast-enhanced and FLAIR-weighted MRI, and the TAC of a patient (patient #6) with an IDH-wildtype anaplastic astrocytoma (WHO grade III) and prognostically unfavorable static and dynamic FET PET parameters (MTV = 41.0 ml; TBR_mean_ = 2.2; TTP = 13 min). The patient had an unfavorable outcome with a PFS of 6.7 months and an OS of 14.0 months.

ROC analyses revealed that the static FET PET parameter TBR_max_ predicted a favorable PFS of ≥ 7.0 months with a sensitivity of 90% and a specificity of 75% (AUC, 0.78 ± 0.12; threshold, 2.0; *P* = 0.050). Additionally, the best prediction of a PFS of 7.0 months or more could be obtained with the static FET PET parameter MTV (sensitivity, 80%; specificity, 88%; AUC, 0.88 ± 0.09; threshold, 14.8 mL; *P* = 0.009) (Fig. 1). In contrast, dynamic FET PET parameters were not prognostic for a favorable PFS of ≥ 7.0 months. Neither static nor dynamic FET PET parameters predicted an OS of ≥ 15.0 months.

Concerning MRI metrics, ROC analyses revealed that volumes of contrast enhancement (threshold, 0.1 mL for both PFS and OS) and the FLAIR signal (thresholds, 22.1 mL and 36.2 mL for PFS and OS, respectively) were not prognostic for a favorable PFS or OS (*P* > 0.05). Supplementary Tables 1 and 2 provide a summary of the ROC analyses results.

### Univariate survival analysis

**Figure 2:**
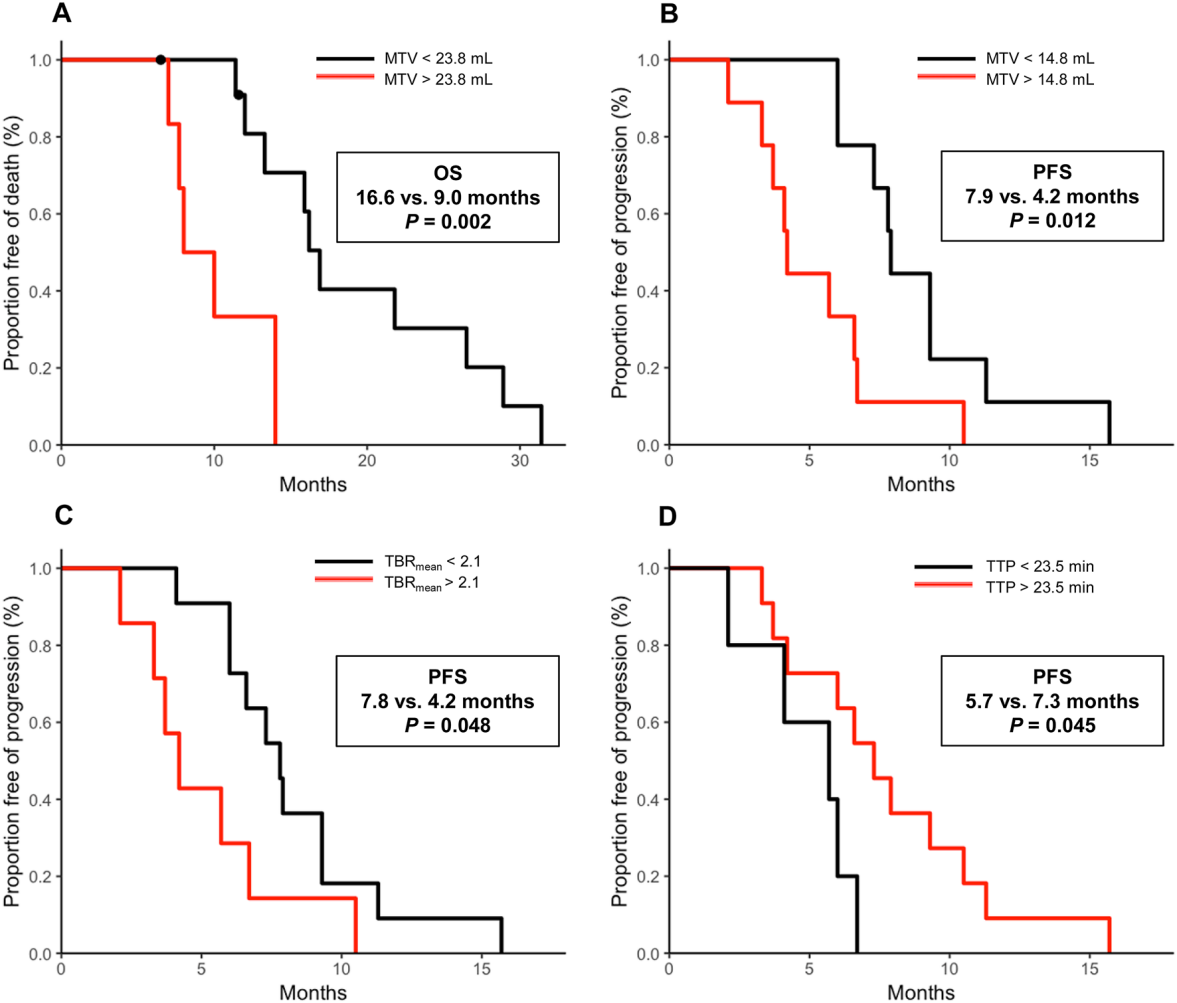
Kaplan–Meier curves for OS and PFS separated by the MTV (**A**,**B**), TBR_mean_ (**C**), and TTP (**D**) within the patient group of newly diagnosed and IDH-wildtype astrocytic glioma.

Patients with a MTV of ≤ 14.8 mL had a doubled PFS (7.9 vs. 4.2 months; *P* = 0.012) (Fig. 2). Likewise, although not reaching a significance level in the ROC analysis, patients with a TBR_mean_ ≤ 2.1 or a TTP ≥ 23.5 min had a prolonged PFS (7.8 vs. 4.2 months and 5.7 vs. 7.3 months; *P* = 0.048 and *P* = 0.045 respectively). Additionally, patients with a MTV of ≤ 23.8 mL had an almost doubled OS (16.6 vs. 9.0 months, *P* = 0.002) (Table 3).

**Table 3:** Results of univariate survival analyses regarding general prognostic factors, FET PET, and MR imaging parameters.

Parameter	Threshold	Univariate PFS analysis		Threshold	Univariate OS analysis	
		*P*-value	PFS (months)		*P*-value	OS (months)
MGMT promoter	Methylated promoter	0.229	6.6 vs. 5.7	Methylated promoter	0.807	16.1 vs. 14.0
Age	≤ 50 vs. > 50 years	0.074	6.0 vs. 7.9	≤ 50 vs. > 50 years	0.343	15.9 vs. 12.0
TBR_max_	2.0	0.231	7.9 vs. 5.7	2.2	0.347	12.7 vs. 15.0
TBR_mean_	2.1	0.048	7.8 vs. 4.2	1.9	0.224	12.4 vs. 15.0
MTV	14.8 mL	0.012	7.9 vs. 4.2	23.8 mL	0.002	16.6 vs. 9.0
TTP	23.5 min	0.045	5.7 vs. 7.3	35.5 min	0.827	11.4 vs. 15.9
Slope	− 0.8 SUV/h	0.062	5.7 vs. 7.3	0.4 SUV/h	0.949	12.7 vs. 16.1
Contrast-enhancing volume on MRI	0.1 mL	0.180	7.9 vs. 6.3	0.1 mL	0.980	16.1 vs. 13.0
Contrast enhancement on MRI	No enhancement vs. enhancement	0.180	7.9 vs. 6.3	No enhancement vs. enhancement	0.980	16.1 vs. 13.0
FLAIR volume on MRI	22.1 mL	0.001	4.0 vs. 7.6	36.2 mL	0.293	16.6 vs. 13.0

*FLAIR* fluid attenuated inversion recovery, *MGMT* O^6^-methylguanine-DNA methyltransferase, *MTV* metabolic tumor volume, *OS* overall survival, *PFS* progression-free survival, *slope* = slope of tracer uptake 20–50 min post-injection*,* *TBR*_*max*_ maximum tumor-to-brain ratio*,* *TBR*_*mean*_ mean tumor-to-brain ratio, *TTP* time-to-peak.

In contrast to FET PET imaging parameters, general prognostic factors, such as MGMT promoter methylation status and age, were not predictive for a prolonged PFS or OS (both *P* > 0.05). About MRI, the contrast-enhancing volume and the presence of any contrast enhancement at all, were not predictive for a prolonged PFS (both 7.9 vs. 6.3 months; *P* = 0.180) or OS (both 16.1 vs. 13.0 months; *P* = 0.980). Whereas the FLAIR volume predicted a significantly longer PFS (threshold, 22.1 mL; 4.0 vs. 7.6 months; *P* = 0.001), it was not predictive for a prolonged OS (threshold, 36.2 mL; 16.6 vs. 13.0 months; *P* = 0.293) (Table 3).

### Multivariate survival analysis

The MTV remained statistically significant (*P* = 0.043; HR, 1.047; 95% CI, 1.002—1.095) in the multivariate survival analysis, indicating an independent prognostic factor for OS. In contrast, age, contrast-enhancing volume on MRI, and MGMT promoter methylation were not significant (all *P* > 0.05) (Table 4).

**Table 4:** Results of multivariate survival analyses.

Parameter	Multivariate PFS analysis				Multivariate OS analysis			
	Threshold	Hazard ratio	95% confidence interval	*P*-value	Threshold	Hazard ratio	95% confidence interval	*P*-value
MTV	14.8 mL	1.011	0.966–1.059	0.635	23.8 mL	1.047	1.002–1.095	0.043
Contrast-enhancing volume on MRI	0.1 mL	1.212	0.750–1.959	0.431	0.1 mL	0.719	0.490–1.055	0.091
Age	50 years	3.599	0.850–15.245	0.082	50 years	0.710	0.205–2.458	0.589
MGMT promoter	methylated	0.786	0.202–3.061	0.729	methylated	0.914	0.254–3.294	0.891

*FLAIR* fluid attenuated inversion recovery, *MGMT* O^6^-methylguanine-DNA-methyltransferase, *MTV* metabolic tumor volume, *OS* overall survival, *PFS* progression-free survival.

## Discussion

The present study’s main finding is that the static FET PET parameter MTV may identify a prognostically more favorable subgroup of patients with newly diagnosed, non-resectable IDH-wildtype astrocytic glioma with minimal or absent MRI contrast enhancement. This prognostic potential similarly applies to the static parameter TBR_mean_ and the dynamic parameter TTP, albeit to a lower significance level. Thus, besides histomolecular features, FET PET-derived imaging parameters may serve as additional prognostically valuable biomarkers. This finding is of immediate clinical relevance in the selected subgroup of glioma patients. The lack of clear contrast enhancement on MRI and the tumor localization in partly deep or eloquent brain areas renders precise neurosurgical targeting more complicated and makes complete resection practically impossible. Combined with the histomolecular characteristics of these tumors, this results in a poor prognosis for affected patients. This underlines the need of early identification of prognostically more favorable patients. Thus, our observations may be of value for patient counseling and affect treatment decisions, with a stronger emphasis on patient-tailored treatment strategies based on both molecular markers and advanced imaging biomarkers such as static and dynamic FET PET. As expected, due to the inclusion of patients without a relevant contrast enhancement on MRI, the contrast-enhancing volume failed to identify patients with a more favorable prognosis. In contrast, the FLAIR volume showed predictive value for PFS. However, this relationship was paradoxical, i.e., patients with higher FLAIR signal volumes exhibited a longer PFS, which is in contrast to the expected clinical course of these patients. Form our view, this relationship was most probably attributed to the small size of this highly selected group of patients, being confirmed by the lack of a prognostic value of the FLAIR volume for OS.

Our results are in line with but extend two earlier studies, which revealed a prognostic value of static pre-irradiation FET PET parameters such as MTV and tumor-to-brain ratios^16,17^. Unlike in our study, in these two studies, gliomas were characterized only by histology according to the WHO classification 2007^1^. In another study by our group^15^, the potential of dynamic FET PET parameters, particularly TTP, to identify patients with a prolonged survival before initiation of chemoradiation was already observed, which is also compatible with the present data. Furthermore, the patients included in our study represent a more homogenous group of only partially resected or biopsied IDH-wildtype astrocytic gliomas with a subtle MRI contrast enhancement at the most.

There are several limitations to our study. False-negative FET PET results may occur in patients with glioma^34^, with adverse effects on prognosis evaluation. On the other hand, earlier studies suggested that in the vast majority, anaplastic gliomas and glioblastomas exhibit increased FET tracer uptake^9,35^. Further limitations are the retrospective nature of the present study and the small number of patients. Nevertheless, it has to be pointed out that the identified glioma subgroup not eligible for complete resection and without a clear and well-defined contrast enhancement is histomolecularly well-characterized and is considered to have an unfavorable prognosis. Further prospective and biopsy-controlled studies with a larger patient cohort are warranted to confirm the FET PET-derived imaging biomarkers’ prognostic value in this patient subgroup.

Taken together, our data suggest that within a neuropathologically defined subgroup of patients with newly diagnosed, not completely resectable IDH-wildtype astrocytic glioma with minimal or absent contrast enhancement on MRI, static and dynamic FET PET parameters have a prognostic value before initiation of chemoradiation. Notably, MTV predicted a prolonged OS independent of other decisive prognostic factors and MRI contrast enhancement. Our data’s remarkable evidence is FET PET-derived parameters’ ability to identify patients with a prolonged survival already before the initiation of chemoradiation. Consequently, FET PET is a clinically valuable method to obtain relevant prognostic information for these patients, justifying its more widespread use.

## Supplementary Information

Supplementary Information.

## Data Availability

All data generated or analyzed during this study are included in this published article and in its supplementary data files.
