# Supplementary material for: Prognostic value of pre-irradiation FET PET in patients with not completely resectable IDH-wildtype glioma and minimal or absent contrast enhancement
Source: Sci Rep. 2021 Oct 21;11:20828. doi: 10.1038/s41598-021-00193-x (PMC8531450; doi:10.1038/s41598-021-00193-x)
Supplement: Supplementary file 1 — Supplementary Information. [file 41598_2021_193_MOESM1_ESM.docx]

**Supplementary Table 1:** Diagnostic performance of static and dynamic PET parameters for predicting a PFS of 7.0 months or more

|  | **TBR_max_** | **TBR_mean_** | **MTV** | **TTP** | **Slope** |
| --- | --- | --- | --- | --- | --- |
| **Threshold** | 2.0 | 2.1 | 14.8 | 23.5 | -0.8 |
| **Sensitivity** | 90% | 60% | 80% | 50% | 50% |
| **Specificity** | 75% | 88% | 88% | 100% | 100% |
| **AUC ± standard deviation** | 0.78 ± 0.12 | 0.75 ± 0.12 | 0.88 ± 0.09 | 0.77 ± 0.12 | 0.66 ± 0.14 |
| ***P*-value** | 0.050 | 0.080 | 0.009 | 0.089 | 0.327 |

**Abbreviations: AUC =** area under the receiver operating characteristic curve; **MTV =** metabolic tumor volume; **PFS** = progression-free survival; **slope** = slope of tracer uptake 20-50 min post-injection; **TBR_max_** = maximum tumor-to-brain ratio; **TBR_mean_** = mean tumor-to-brain ratio; **TTP** = time to peak

**Supplementary Table 2:** Diagnostic performance of static and dynamic PET parameters for predicting an OS of 15.0 months or more

|  | **TBR_max_** | **TBR_mean_** | **MTV** | **TTP** | **Slope** |
| --- | --- | --- | --- | --- | --- |
| **Threshold** | 2.2 | 1.9 | 23.8 | 35.5 | 0.4 |
| **Sensitivity** | 55% | 46% | 63% | 80% | 90% |
| **Specificity** | 57% | 71% | 100% | 50% | 50% |
| **AUC ± standard deviation** | 0.49 ± 0.15 | 0.53 ± 0.15 | 0.69 ± 0.14 | 0.68 ± 0.14 | 0.73 ± 0.14 |
| ***P*-value** | 1.000 | 0.855 | 0.189 | 0.272 | 0.141 |

**Abbreviations: AUC =** area under the receiver operating characteristic curve; **MTV =** metabolic tumor volume; **OS** = overall survival; **slope** = slope of tracer uptake 20-50 min post-injection; **TBR_max_** = maximum tumor-to-brain ratio; **TBR_mean_** = mean tumor-to-brain ratio; **TTP** = time to peak
